# Effects of red clover on hot flash and circulating hormone concentrations in menopausal women: a systematic review and meta-analysis

**Published:** 2015

**Authors:** Masumeh Ghazanfarpour, Ramin Sadeghi, Robab Latifnejad Roudsari, Khadijeh Mirzaii Najmabadi, Mojtaba mousavi bazaz, Somayeh abdolahian, Talat Khadivzadeh

**Affiliations:** 1 *Student Research Committee, Department of Midwifery, School of Nursing and Midwifery, Mashhad University of Medical Sciences, Mashhad, Iran*; 2 *Nuclear Medicine Research Center, Mashhad University of Medical Sciences, Mashhad, Iran*; 3 *Evidence-Based Care Research Centre, Department of Midwifery, School of Nursing and Midwifery, MashhadUniversity of Medical Sciences, Mashhad, Iran*; 4 *Department of Midwifery, School of Nursing and Midwifery, Mashhad University of Medical Sciences, Mashhad, Iran*; 5 *Department of community of Medicine, School of Medicine, Mashhad University of Medical Sciences, Mashhad, Iran*; 6 *Department of Midwifery, Islamic Azad University, Firuzabad, Fars, Iran *

**Keywords:** *Red clover*, *Hotflashes*, *Circulating hormone concentrations*, *Menopause*, *Systematic review*, *Meta-analysis*

## Abstract

**Objective:**

To critically evaluate the effect of red clover on hot flash, endometrial thickness, and hormones status in postmenopausal and peri- and post-menopausal women.

**Materials and Methods:**

MEDLINE (1966 to July 2014), Scopus (1990 to July 2014), and the Cochrane Central Register of Controlled Trials (The Cochrane Library issue 1, 2014) were searched for published randomized controlled Trials (RCTs).

**Results:**

Of 183 relevant publication trials, 11 RCTs met the inclusion criteria. The mean hot flashes frequency in red clover was lower than the control groups (MD -1.99; p=0.067). There was larger decrease in FSH (SMD -0.812; CI: -1.93 to 0.312; p=0.157) and SHBG (SMD -0.128; CI-0.425 to 0.170; P=0.4) in red clover group, compared with placebo, which was not however statistically significant. LH (SMD 0.144; CI-0.097 to 0.384, p=0.242), estradiol (SMD 0.240; CI-0.001 to 0.482, p=0.051), testosterone (MD 0.083; CI: -0.560 to 0.726; p=0.901), and endometrial thickness (SDM 0.022; CI: -0.380 to 0.424, p=0.915) showed greater increase in red clover, compared with placebo, although the effect of estradiol was only significant.

**Conclusion:**

Red clover had a positive effect of alleviating hot flash in menopausal women. Our data, however, suggested very slight changes in FSH, LH, testosterone, and SHBG and significant effect in estrogen status by red clover consumption. However, the interpretation of results of the current study is limited due to methodological flaws of the included studies, menopause status, and large heterogeneity among them. Further trials are still needed to confirm the current finding.

## Introduction

Menopause is defined as amenorrhea for at least 12 months consecutively (Kotsopoulos, et al., 2000 ▶). It is characterized by remarkable hormonal and often social-psychological changes. Hormone therapy was widely recommended to some women for alleviation of menopausal symptoms (MacLennan et al., 2004 ▶, Loprinzi et al., 2000 ▶, Hidalgo et al., 2005 ▶). Despite this, compliance was low in both developed and developing countries (Hidalgo et al., 2005 ▶). In Iran, a developing country, percentage of menopausal women who uses hormone replacement therapy (HRT) is 15%, while only 8.75% of users continue the treatment (Menati et al., 2014 ▶). The common causes that lead to discontinuation of HRT are adverse effects, fear of inducing cancer, vaginal bleeding, and financial problems (Hidalgo et al., 2005 ▶). Misinformation of the findings of the first Women’s Health Initiative (WHI) has also led to further decrease in use of HRT (Gompel and Santen, 2012 ▶, Meherishi et al., 2010 ▶), despite an updated analysis which showed HRT did not increase the risk of breast cancer (Safajou et al., 2014).

There has been a growing interest in red clover extract derived isoflavones among women according to scientific literature (Lipovac et al., 2011 ▶). The effect of red clover has been comprehensively assessed in several systematic reviews and showed a range from weak beneficial effect (Krebs et al., 2004 ▶, Lethaby et al., 2007 ▶, Nelson et al., 2006 ▶) to significant effect (Thompson Coon et al., 2007). Some animal studies have raised concern regarding high dose of red clover intake and an increased risk of estrogen-dependent cancers (Sites et al., 2014 ▶). Aim of this systematic review and meta-analysis was to assess the effect of red clover on hot flash, endometrial thickness, estradiol concentration, and other hormones profiles.

## Material and Methods

MEDLINE (1966 to July 2014), Scopus (1990 to July 2014), and the Cochrane Central Register of Controlled Trials (The Cochrane Library issue 1, 2014) were searched for published randomized controlled trials (RCTs). Search keywords were “menopause AND (red clover, trifolium pratense, cow clover, meadow clover, purple clover, beebread, trefoil”. No language limit was imposed on the search. We also searched Persian databases (SID, Iran medex, Magiran, Medlib, Iran doc, and Google Scholar) using equivalent keywords in July 2014. In addition, reference section of relevant trials, systematic review and meta-analysis were manually checked to identify further trials missed by electronic search and the authors were contacted by email to obtain additional data but no answer was received and this may be considered as a limitation of this review. Possibility of bias was assessed by funnel plot and Egger's tests. Moreover, sensitive analysis was performed to assess the influence of excluding each study on heterogeneity.

**Inclusion criteria**

Trials were included systematic review if they reported the following criteria

(1) Include peri- and post-menopausal or post menopause women with complaints of hot flashes. (2) Was parallel-group or crossover RCT.

(3) Compared oral red clover as the mono-preparations in the intervention arm were included regardless of the control group type.

**Primary outcomes**

*Vasomotor*

1) Hot flashes frequency, 2) Hot flashes intensity, 3) Sweating at nights

**Secondary outcomes**

1) The effects of red clover on hormonal status namely follicle stimulating hormone (FSH), luteinizing hormone (LH), Sex hormone-binding globulin (SHBG), estradiol, testosterone were evaluated. 2) The effect of red clover on endometrial thickness.

**Figure 1:**
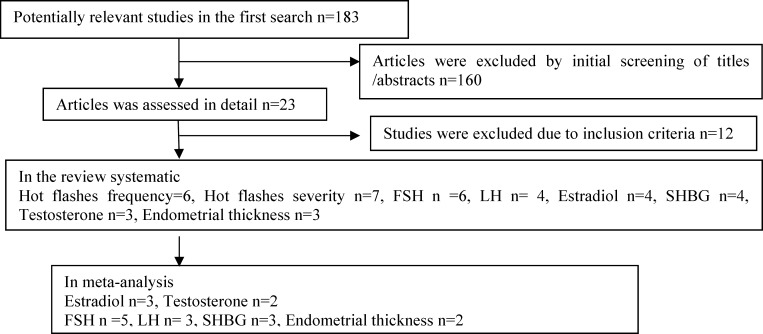
Search strategy of the study

**Data extraction**

We estimated the difference between means in two ways: difference in means (MD) and standardized difference in means (SMD). The latter was used when studies included in the meta-analysis measured same outcome by different measurement units. Changes in mean (frequency of hot flashes, FSH, LH, testosterone, and SHBG estrogen) at baseline and endpoint were assessed. 

For each study, we extracted the following data according to a pre-defined checklist: first author, menopause status, age, study design, study duration, sample size, and quality of trials. The latter was assessed by two reviewers using oxford center for evidence based medicine checklist for therapeutic studies. Data were independently assessed by two authors and disparities were resolved by discussion with a third researcher. Agreement between raters of the two reviewers was assessed using the kappa (K) statistic (Cohen 1968). Overall, there was complete agreement between the two reviewers.

**Quality assessment of the included studies**

The quality of the included studies was evaluated by Oxford Center for Evidence Based Medicine checklist for RCTs.

**Statistical analyses**

We interpreted the results using random effects model (Der-simonian and Laird method) because of presence of large heterogeneity among included trials in our meta-analysis.

For heterogeneity evaluation, Cochrane Q test (p<0.05 as statistically significant) and I^2^ index were used. Latter was used to assess how much of the variance across studies is likely to be real and is not due to sampling errors. 

All statistical analyses were done by Comprehensive Meta-analysis Version 2 (Biostat, Englewood, NJ, USA). 

## Results

Of 183 relevant publication trials, 11 RCTs met the inclusion criteria. The summarized characteristics of the included studies are shown in Table 1.

**The effect of red clover on the frequency of hot flashes**

*Red clover versus placebo*

In our pervious meta-analysis (Ghazanfarpour et al., in press), 6 trials, Showed that a greater decline in the red clover-treated patients as compared to the placebo group (with marginal statistical significance). MD was -1.99; p=0.067)(Baber et al., 1999 ▶, Lipovac et al., 2011 ▶, Tice et al., 2003 ▶, van de Weijer and Barentsen, 2002 ▶, Knight et al., 1999 ▶, Jeri et al., 2002 ▶).

**Table 1:** Characteristics of 11 randomized placebo-controlled trials included in our systematic review

**Author, Year**	** Duration ;** **Week**	**Age;Year**	**Design**	**Status** **menopause**		**FSH** **mIU/ml**	**length of amenorrhea (months)**	**Isoflavone/mg**	**Participants** **Intervention**	** Participants** ** control**		**blinding method**
**Jeri, 2002**	16	52	P	Post		> 30	>12	40	14		9	Yes
**Atkinson, 2004**	51	55	P	Peri-and post		> 30	>12	40	86		91	Yes
**Lipovac, 2012**	12×2 (wash out=1)	53	Co	Post		>35	>12	80	50		59	Yes
**Baber, 1999**	12×2 (wash out=4)	54	Co	Peri-and post		>30	>6	40	43		44	Yes
**Knight, 1999** **.**	12	54	P	Peri-and post		>40 U/l	≥ 6	40	12		12	Yes
**Geller, 2009**	12	52	P	Peri-and post		>40	>12	120	14		17	Yes
**van de Weijer, 2002**	12	53	P	Post		Not known	>12	80	15		11	Yes
**Tice, 2003**	12	52	P	Peri-and post		>30	>6	82	84		85	Yes
**Giorno, 2010**	24	53	P	Post		> 30	>12	40	50		50	Yes
**Ehsanpour, 2012**	10	53	P	post		Not mentioned	12>	45	28		27	Yes
**Hidalgo, 2005**	12×2(wash out=1)	51	Co	Post	>30		12>	80	53		53	Yes

Abbreviations; Post: post menopause; Peri: perimenopausal; Co: cross over; P: parallel; FSH: follicle stimulatin hormone

**Figure 2:**
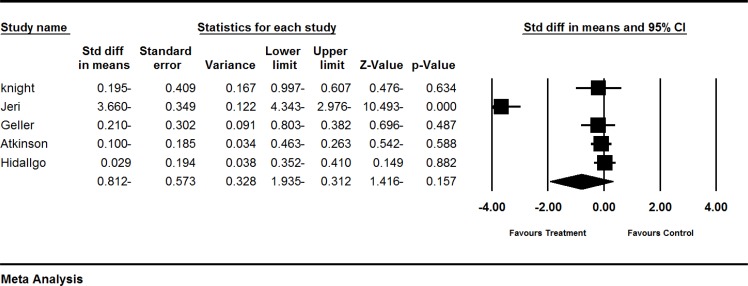
Effects of red clover on FSH. The horizontal lines denote the 95% CI, ■ point estimate (size of the square corresponds to its weight); ♦, combined overall effect of treatment

*Red clover vs. black cohosh*

Only one trial (Geller et al., 2009 ▶) compared red clover and black cohosh. Red clover and black cohosh arms showed 51% and 34% decrease in hot flashes frequency, respectively. The effect size however was not statistically significant: MD=-10.24 (-26.21 to 5.73; p=0.209; 32 women). 

*Red clover vs. HRT*

Only one trial (Maki et al., 2009 ▶) compared red clover and an HRT arm. Red clover and HRT arms showed 51% and 93% decrease in hot flashes frequency, respectively. The effect size was statistically significant: MD=-36.74 (8.44 to 65.03 p=0.011; 31women).

**The effect of red clover on hot flashes intensity**

Seven trials (del Giorno et al., 2010 ▶, Ehsanpour et al., 2012 ▶, Hidalgo et al., 2005 ▶, Geller et al., 2008 ▶, Salehi et al., 2013a ▶, Taavoni et al., 2012 ▶, Jeri et al., 2002 ▶) investigated the effect of red clover on the intensity of hot flashes. One trial by Jeri found a statistically significant decrease (47%) in red clover group, while changes remained close to baseline in the control group. The published trial did not report any comparisons between groups.

In the second trial by Hidalgo et al. (Hidalgo et al., 2005 ▶), percentage of symptomatic patients reporting hot flash intensity, decreased significantly (85%) in red clover as compared to the placebo group (2%), which was statistically significant (p<0.05).

Third trial by Ehsanpour et al. (Ehsanpour et al., 2012 ▶) showed that the percentage of symptomatic patients reporting hot flashes, decreased significantly in the red clover compared to the placebo group (p=0.04).

Fourth trial by Geller et al. (Geller et al., 2008 ▶) assessed hot flash intensity for four time points: at 3, 6, 9, and 12 months. Reduction in intensity of hot flashes was the same in both control and treatment group up to 3 months but less effectiveness was observed between 3 and 9 for red clover group. Effectiveness again was the same in the end week of study. The Comparison of two groups was not significant in the end week of study.

Fifth trial by Girona et al. (del Giorno et al., 2010 ▶) showed statistically significant improvement in both red clover (58%) and placebo groups (62%) at 12 weeks. Difference between groups was not statistically significant. However, two trials (del Giorno et al., 2010 ▶, Geller et al., 2008 ▶) were not consistent with other trials. It is important to emphasize that both trials had a strong placebo effect, which obscured the actual efficacy of red clover. 

Sixth trial by Salehi et al. (Salehi et al., 2013b ▶) assessed the effect of red clover on hot flash intensity. According to Friedman test Friedman test, the frequency of mild, moderate, and severe hot flash decreased significantly compared to the baseline in both red clover (p<0.001) and placebo group (p<0.001). Mann–Whitney test showed a statistically significant decrease between groups at week 10 of the study (p=0.04), but it was not significant at weeks 2 and 4.

Finally, Taavoni et al. (Taavoni et al., 2012 ▶) compared two groups of red clover and placebo finding. The severity of vasomotor symptoms decreased from 2.28±1.30 to 1.42±0.96 in red clover compared to a decrease 2.33±1.28 to 2.11±1.0 in control groups. Comparison of two groups was statistically significant (p=0.003)

**Night sweating**

Two trials (Hidalgo et al., 2005 ▶, Lipovac et al., 2012 ▶) assessed the effect of red clover on the night sweating. In the trial by Hidalgo et al. (Hidalgo et al., 2005 ▶), percentage of symptomatic patients reporting the night sweat decreased (from 96.2% to 30.2%) in the red clover group as compared to the placebo group (96.2% to 92.2%), and the difference between groups was statistically significant (p<0.05).

In the trial by Lipovac et al. (Lipovac et al., 2011 ▶), night sweat daily frequency decreased by 73% in the red clover group while remained close to baseline in the placebo group. The comparison of the red clover group and placebo using Wilcoxon rank test also showed statistically significant improvement in the night sweat daily frequency in the patients treated with red clover (p=0.0001). 

**The effect of red clover on** **hormonal status of the patients **

*The effect of red clover on FSH levels*

Six trials (Atkinson et al., 2004 ▶, Geller et al., 2008 ▶, Hidalgo et al., 2005 ▶, Imhof et al., 2006 ▶, Jeri et al., 2002 ▶, Knight et al., 1998 ▶) assessed the effect of red clover on FSH levels in postmenopausal and peri- and post-menopausal women. The SMD of FSH levels showed greater decrease in the red clover group as compared to the control groups (pooled MD -0.812; 95% CI: -1.93 to 0.312; p=0.157; heterogeneity p<0.00001; I^2^=96%; 379 women; random effects model; 5 trials), which, was not significant (Atkinson et al., 2004 ▶, Geller et al., 2008 ▶, Hidalgo et al., 2005 ▶, Jeri et al., 2002 ▶, knight., 1999 ▶). The forest plot is shown in Figure 2.

**Figure 3:**
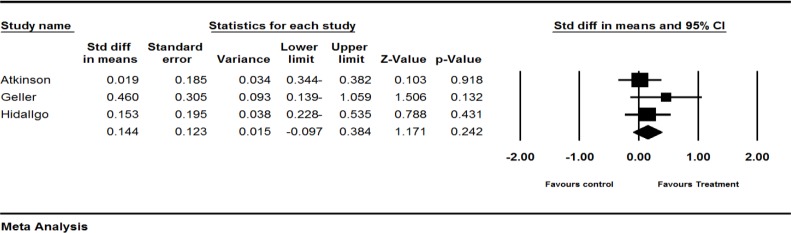
Effects of red clover on LH. The horizontal lines denote the 95% CI, ■ point estimate (size of the square corresponds to its weight); ♦, combined overall effect of treatment

**Table 2:** Subgroup analyses of the effects of red clover based on menopause status.SMD, Standardized difference in means; CI, confidence interval

**Variable**	**Number of RCTs**	**Sample size treatment** **/control)**	**p-value for heterogeneity **	**p-value**	**Random effect model** **SMD (95% CI)**
**FSH Levels**					
**Post-menopause (Hidalgo et al., 2005, Jeri et al., 2002)**	2	95/99	p=0.000 I^2^=98%	0.328	-1.80 (-5.41 to 1.81)
**Peri- and post-menopausal (knight., 1999, Geller et al., 2008, Atkinson et al., 2004)**	3	93/95	p=0.756 I^2^=0%	0.374	-0.139 (-0.427to 0.150)
**Test for subgroup difference**					0.309
**LH Levels**					
**Post-menopause (Hidalgo et al., 2005)**	1	53/53	--------	0.431	0.153 (-0.22 3to 0.535)
**Peri- and post-menopausal (Atkinson et al., 2004, Geller et al., 2008)**	2	78/83	I^2^=0%	0.411	0.173 (-0.239 to 0.58)
**Test for subgroup difference**					0.256
**Estradiol levels **					
**Post-menopause (Hidalgo et al., 2005)**	1	53/53		0.203	-0.24 (-0.630 to 0.134)
**Peri-** **and post-menopausal (Atkinson et al., 2004, Geller et al., 2008)**	2	78/83	p=0.374 I^2^=0%	0.138	0.235 (-0.075 to 0.546)
**Test for subgroup difference**					0.727
**SHBG levels**					
**Post-menopause (Hidalgo et al., 2005)**	1	53/53	--------	0.623	-0.096 (-0.477 to 0.285)
**Peri- and post post-menopausal (Geller et al., 2008, Knight et al., 1999)**	2	34/34	p=0.925 I^2^=0%	0.465	-0.178 (-0.65 to 0.299)
**Test for subgroup difference**					
**Testosterone levels**					0.290
**Post-menopause (Hidalgo et al., 2005)**	1	53/53	--------	0.290	-0.206 (-0.583 to 0.176)
**Peri- and post-menopausal (Geller et al., 2008)**	1	22/22	--------	0.136	0.455 (-0.143 to 1.054)
**Test for subgroup difference**					0.928

The fifth, trial by Imhof et al. (Imhof et al., 2006 ▶) was not included in our quantitative analysis due to incomplete reporting. FSH increased (6.54 mlU/ml) compared to an increase (1.76 mlU/ml) in placebo group, which was marginally significant (p=0.069). High heterogeneity was observed in the trial (I^2^=98%). To further explain this heterogeneity, we performed sub-group and sensitive analyses. The results of sub-group analyses according to menopausal status demonstrated that heterogeneity decreased to 0% in trials performed on peri- and post-menopausal women. The result of subgroup analysis based on menopausal status did not show any statistically significant difference between subgroups (p=0.309) table 2.

*The effect of red clover on LH level*

Four trials (Atkinson et al., 2004 ▶, Geller et al., 2008 ▶; Hidalgo et al., 2005 ▶, Imhof et al., 2006 ▶) assessed the effect of red clover on LH level in postmenopausal and peri- and post-menopausal women. The SMD of LH levels was larger in the red clover group, compared to the control groups (pooled SMD 0.144; 95% CI-0.097 to 0.384, p=0.242; heterogeneity p=0.466; I^2^=0%; 267 women; random effects model; 3 trials) (Atkinson et al., 2004 ▶; Geller et al., 2008 ▶, Hidalgo et al., 2005 ▶), which was not statistically significant. The forest plot is shown in Figure 3.

The fourth trial by Imhof et al. (Imhof et al., 2006 ▶) was not included in our quantitative analysis due to incomplete reporting. A greater decline in placebo (−2.45) was observed as compared to red clover group (−0.43), which was not statistically significant (p=0.861). 

The result of subgroup analysis based on menopausal status was not significant (p=0.256). The result is illustrated in Table 2.

*The effect of red clover on estradiol levels*

**Figure 4:**
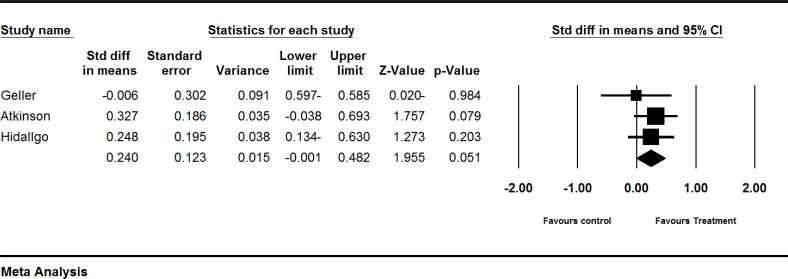
Effects of red clover on estradiol. The horizontal lines denote the 95% CI, ■ point estimate (size of the square corresponds to its weight); ♦, combined overall effect of treatment

Four trials (Atkinson et al., 2004 ▶; Geller et al., 2008 ▶; Hidalgo et al., 2005 ▶; Imhof et al., 2006 ▶) assessed the effect of red clover on postmenopausal and peri- and post-menopausal women. The SMD of estradiol levels was larger in the red clover group as compared to the control groups (pooled SMD 0.240; 95% CI-0.001 to 0.482, p=0.051; heterogeneity p=0.101; I^2^=56%; 267 women; random effects model; 3 trials) (Atkinson et al., 2004 ▶; Geller et al., 2008 ▶; Hidalgo et al., 2005 ▶). The forest plot is shown in Figure 4. The fourth trial by Imhof et al. (Imhof et al., 2006 ▶) was not included in our quantitative analysis due to incomplete reporting. Their study showed an increase (1.85 pg/ml) in red clover group as compared to a decrease in placebo group (−5.87 pg/ml), which was not statistically significant (p= 0.861). The result is illustrated in Table 2.

*The effect of red clover on SHBG levels*

**Figure 5:**
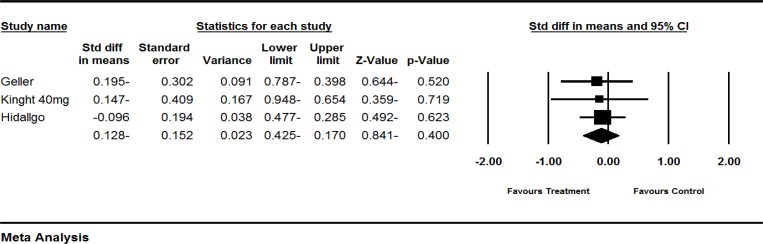
Effects of red clover on SHBG. The horizontal lines denote the 95% CI, ■ point estimate (size of the square corresponds to its weight); ♦, combined overall effect of treatment

Four trials (Geller et al., 2008 ▶; Knight et al., 1999 ▶; Hidalgo et al., 2005 ▶; Imhof et al., 2006 ▶) assessed the effect of red clover on SHBG levels in postmenopausal and peri- and post-menopausal women. SHBG levels showed greater decrease in red clover-treated patients as compared to the control groups (pooled standardized MD -0.128 95%; CI -0.425 to 0.170; p=0.4; heterogeneity p=0.962; I^2^=0%; 174 women; random effects model, 3 trials), which was not statistically significant (Geller et al., 2008 ▶, Hidalgo et al., 2005 ▶, Knight et al., 1999 ▶). The forest plot is shown in Figure 5. 

 One trial (Imhof et al., 2006 ▶) was not included in our quantitative analysis due to incomplete reporting. Imhof et al. (Imhof et al., 2006 ▶) showed a greater decrease in the red clover group (-3.70 nmol/l) as compared to the placebo group (-3.58 nmol/l), which was statistically non-significant (p= 0.824). The result of subgroup analysis based on menopausal status did not show any statistically significant difference between subgroups (p=0.290). The result is illustrated in Table 2.

*The effect of red clover on testosterone levels*

**Figure 6:**
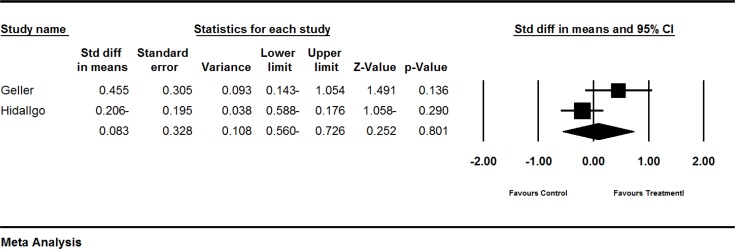
Effects of red clover on testosterone. The horizontal lines denote the 95% CI, ■ point estimate (size of the square corresponds to its weight); ♦, combined overall effect of treatment

Three trials (Geller et al., 2008 ▶; Hidalgo et al., 2005 ▶; Imhof et al., 2006 ▶) assessed the effect of red clover on testosterone levels in postmenopausal and peri- and post-menopausal women. Testosterone levels showed greater increases in red clover-treated patients, compared to the control groups (pooled MD 0.083; 95% CI: -0.560 to 0.726; p=0.901; heterogeneity p=0.068; I^2^=69%; 150 women; random effects model; 2 trials) (Geller et al., 2008 ▶, Hidalgo et al., 2005 ▶., which was not statistically significant. The forest plot is shown in Figure 6.

The third trial by Imhof et al. (Imhof et al., 2006 ▶) was not included in our quantitative analysis due to incomplete reporting. A greater increase was observed in red clover (0.12 ng/ml), as compared to placebo (-0.02) in testosterone levels, which was statistically significant (p=0.003).

 The result of subgroup analysis based on menopausal status did not show any statistically significant difference between subgroups (p=0.928).

*The effect of red clover on endometrial thickness*

**Figure 7:**
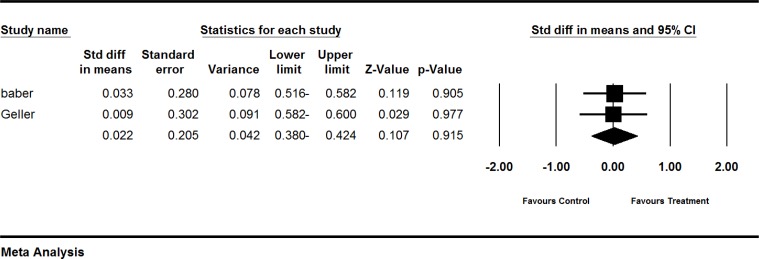
Effects of red clover on endometrial thickness. The horizontal lines denote the 95% CI, ■ point estimate (size of the square corresponds to its weight); ♦, combined overall effect of treatment

In the peri- and post-menopausal women, endometrial thickness showed a greater very slight increase in red clover group as compared to the placebo group (SMD=0.022; 95% CI: -0.380 to 0.424, p=0.915; heterogeneity p=0.952; I^2^=0%; 95 women random effects model; 2 trials) (Baber et al., 1999 ▶; Geller et al., 2008 ▶). The forest plot is shown in Figure 7. Based on only one trial of 92 post-menopausal women, endometrial thickness decreased −0.55 mm (p<0.001) in red cover group compared to a decrease of −0.18 mm (p=0.145) in control groups. Comparison of two groups were statistically significant (p=0.001((Imhof et al., 2006 ▶).

.

## Discussion

To the best of our knowledge, this is first systematic review and meta analysis to assess the effect of red clover on hormonal status. Red clover had a positive effect of alleviating hot flash in menopausal women. The meta analysis also showed very slight change regarding FSH, LH, testosterone, and SHBG and significant effect in estrogen status by red clover-derived isoflavones.

**The effect of red clover on the frequency hot flashes**

*Red clover versus placebo*

In our pervious meta-analysis (Ghazanfarpour et al., in press), 6 trials, Showed that a greater decline in the red clover-treated patients as compared to the placebo group (with marginal statistical significance (p=0.067).

**The effect of red clover on hot flashes intensity**

Seven trials (del Giorno et al., 2010 ▶, Ehsanpour et al., 2012 ▶, Hidalgo et al., 2005 ▶, Geller et al., 2008 ▶, Salehi et al., 2013a, Taavoni et al., 2012, Jeri et al., 2002) investigated the effect of red clover on the intensity of hot flashes. It seems that red clover is effective in the reduction of hot flashes intensity; however more studies with consistent statistical analysis of the effect sizes are needed.

**The effect of red clover on Night sweating**

*Night sweating*

Two trials (Hidalgo et al., 2005 ▶; Lipovac et al., 2012) assessed the effect of red clover on the night sweating and showed significant effect in red clover group in comparison with control group. The mechanism by which red clover-derived isoflavones decreased hot flashes and night sweating has not been assessed. As a matter of fact, current understanding of the etiology of hot flashes has yet remained unclear, although, some studies have demonstrated that most hot flashes are preceded by raising core body temperature (Freedman and Blacker, 2002 ▶). Results of a cited–animal study indicated that intake of phytoestrogens changed the neuroendocrine mechanism of core body temperature regulation (Bu and Lephart, 2005 ▶) which may play a role in decreasing hot flashes.

**The effect of red clover on endometrial thickness**

Three trials (Baber et al., 1999 ▶; Geller et al., 2008 ▶; Imhof et al., 2006) assessed the effect of red clover on the endometrial thickness. Overall, it seems that red clove showed a range of null effect to non-significant decrease in postmenopausal period. A trial by Clifton-Bligh et al. (Clifton-Bligh et al., 2001 ▶), which was not included in the current systematic review due to lack of control group. This study assessed the effect of different dose of red clover (28.5, 57, and 85.5 mg/day) on endometrial thickness. No significant increase was observed on endometrial thickness with any of the doses of isoflavones. Another trial (Hale et al., 2001 ▶) was performed to assess the effect of red clover on Ki-67 proliferative marker of endometrium. Red clover-derived isoflavones showed anti-proliferative effect on endometrial thickness. Further studies with control group are required to confirm this data.

**The effect of red clover on** **LH level**

Four trials (Atkinson et al., 2004 ▶; Geller et al., 2008 ▶; Hidalgo et al., 2005 ▶; Imhof et al., 2006) assessed the effect of red clover on LH levels. To sum up, it was shown that red clover led to a small increase in LH levels as illustrated in Figure 3. 

**The effect of red clover on SHBG levels**

Four trials (Geller et al., 2008 ▶, Knight et al., 1999, Hidalgo et al., 2005 ▶, Imhof et al., 2006) assessed the effect of red clover on SHBG. To sum up, red clover may slightly decrease SHBG levels. Further studies are needed to clarify this effect of red clover on SHBG levels. The result is shown in Figure 5. 

**The effect of red clover on testosterone levels**

Three trials (Geller et al., 2008 ▶, Hidalgo et al., 2005 ▶, Imhof et al., 2006) assessed the effect of red clover on testosterone levels. To sum up, red clover may increase testosterone levels. Further studies are needed to clarify this effect of red clover on testosterone levels. The result is shown in Figure 6. 

**The effect of red clover on FSH levels**

Six trials (Atkinson et al., 2004 ▶, Geller et al., 2008 ▶, Hidalgo et al., 2005 ▶, Imhof et al., 2006, Jeri et al., 2002, Knight et al., 1998) assessed the effect of red clover on FSH levels. Overall, based on current findings, no consistent conclusion can be made and further trials are needed in this regard (Figure 2). 

**The effect of red clover on estradiol levels**

Four trials assessed (Atkinson et al., 2004 ▶; Geller et al., 2008 ▶; Hidalgo et al., 2005 ▶; Imhof et al., 2006) assessed the effect of red clover on estradiol levels. Overall, based on these four trials, red clover may cause significant increase in estradiol levels (Figure 4). 

**Cancer-promoting effects**

Our finding suggests that red clover consumption may have breast cancer-promoting effects. A meta-analysis showed a positive relationship between levels of estradiol and increased risk of breast cancer (Endogenous Hormones and Breast Cancer Collaborative Group, 2002). Our study showed that red clover may increase the risk of estrogen-dependent cancers as estradiol showed a borderline increase in the red clover groups in comparison with control group base on three trials. 

**Cancer-protecting effects**

There is evidence that red clover consumption may protect against the development of estrogen-dependent cancers. A study showed a reverse relationship between levels of urinary isoflavonoids and risk of breast cancer (Zheng et al., 1999). We found that level of urinary isoflavones showed a statistically significant increase in red clover as compared to placebo (pooled standardized MD 1.106; 0.577 to 1.635; p<0.001; heterogeneity p=0.206; I2=36%), based on 3 trials (van de Weijer and Barentsen, 2002; Knight et al., 1999; Baber et al., 1999 ▶). 

Some studies consider breast density as a marker of breast cancer risk (Habel et al., 2004 ▶; Atkinson et al., 2004 ▶). Only one of the included studies in our systematic review evaluated the effect of red clover on mammographic density which showed significant changes in mammographic density in both placebo and red clover groups. However, no difference was observed between groups (Atkinson et al., 2004 ▶) and this cannot be attributed to anti-estrogen or anti-cancer effects of red clover. This effect is most likely due to natural changes of breast density over time because breast density in women receiving red clover group decreased as that of the control group. Another evidence in support of natural changes came from Boyd study. It demonstrated an inverse relation between breast density and age (Atkinson et al., 2004 ▶). Our finding showed a statistically significant decrease in endometrial thickness in post-menopausal women following consumption of red clover, based on only one trial (Imhof et al., 2006). None of the cancer promoting or protecting effects of the red clover can be confirmed consistently by the results of our systematic review and further studies are still needed in this regard.

**Suggestion for future research**

The improvement in the menopausal symptoms in some of the included trials (Baber et al., 1999 ▶; Knight et al., 1999) in placebo group may be due to inadvertent or deliberate consumption of isoflavone-rich food in the placebo group. One direction for future research is to conduct studies that measure urinary excretion of isoflavone as a reflection of inadvertent or deliberate consumption of isoflavone in the placebo group and then comparison of the level of isoflavones between baseline and end point. If change is significant, the interpretation of the result should be considered with caution because of the possibility of bias in the included studies 

Peri- and post-menopausal women with various hormonal statuses were included in most of the studies. One direction for future research is to conduct studies that divide women into two groups based on peri- or post-menopausal status; then each group being further divided into several subgroups according to their circulating levels of estradiol and BMI on which the effect of isoflavonoids seems to depend.

**Limitations of this meta-analysis**

Although we reported the result using random effects model, a large heterogeneity was present among trials. It can be attributed to isoflavones bioavailability, variability between individuals, amount of administered red clover, menopausal status, and variability of red clover and the isoflavones received by other food-sourced. Quality of almost all included studies to the systematic review was also suboptimal. This can decrease the reliability of our results. Future trials should base their design on CONSORT guideline in order to increase the quality. 

## Conclusion

Red clover had a positive effect on alleviating hot flash in menopausal women. Our data, however, suggest very slight changes in FSH, LH, testosterone, and SHBG and significant effect in estrogen status by red clover consumption. However, the results of the current study are limited due to methodological flaws of the included studies, menopause status, and large heterogeneity among them. Further trials are still needed to confirm the current finding.
